# Assessing Telemedicine Competency Among Doctors in a Tertiary Care Hospital: A Questionnaire-Based Cross-Sectional Study

**DOI:** 10.7759/cureus.57712

**Published:** 2024-04-06

**Authors:** Shaik Mabu Shareef, Bhanu Prakash Goud, Bhavika Domalapally, Tadvi Naser Ashraf

**Affiliations:** 1 Department of Pharmacology, Dr. YSR Government Medical College, Pulivendula, IND; 2 Department of Pharmacology, Kurnool Medical College, Kurnool, IND; 3 Department of Pharmacology, Government Medical College, Nalgonda, IND

**Keywords:** health informatics, medical training, doctor's attitude, healthcare technology, telemedicine

## Abstract

Background

The use of telemedicine in contemporary healthcare has become essential, providing a novel method of delivering care, particularly in rural and underdeveloped areas. This study assesses the telemedicine awareness, knowledge, attitude, skills, and challenges among physicians working in tertiary care hospitals.

Methods

A cross-sectional study was carried out with 100 doctors from diverse specialties at a tertiary care institution. The questionnaire evaluated five domains: telemedicine awareness and knowledge, telemedicine attitude, telemedicine technology skills, telemedicine utilization patterns, and perceived barriers and educational needs.

Results

The study indicated that 95% of participants were aware of telemedicine. However, knowledge gaps remained, particularly in legal and ethical concerns (50%) and international rules (40%). Eighty percent of respondents had a favorable opinion of telemedicine, and 85% thought it might improve patient care in rural regions. The proficiency level of telemedicine users was variable: 60% of them had previous experience, and 70% of them rated their proficiency as intermediate or better. According to utilization patterns, 50% of telemedicine users used it at least once a week, primarily for remote monitoring (30%) and video consultations (60%). The study found that 90% of respondents had a high demand for training and educational opportunities. The absence of infrastructure (65%), worries about data security (55%), and patient acceptability (30%) were the main obstacles found. It also highlighted how important it is to have defined policies and collaborate across disciplines.

Conclusion

The study identifies a good attitude toward telemedicine among doctors as well as a need for improved training and infrastructure. It is essential to tackle these requirements and obstacles in order to successfully incorporate telemedicine into healthcare systems.

## Introduction

The emergence and integration of digital technology have radically revolutionized the healthcare sector, with telemedicine emerging as a crucial innovation [1]. It has been acknowledged that telemedicine, or the remote provision of medical services via telecommunications technology, has the potential to increase access to treatment, particularly in rural and underserved areas [2]. The purpose of this study is to assess the tertiary care hospital doctors’ awareness, knowledge, attitude, skills, and obstacles related to telemedicine.

Telemedicine has received extraordinary attention and urgency in response to global health issues and the rising demand for accessible healthcare [3]. It gives medical professionals a platform to communicate with patients, keep an eye on their health from a distance, and deliver care without being limited by geographic location [4]. However, a number of variables, including the preparedness and ability of medical professionals to adjust to these technological improvements, are critical to the successful use of telemedicine [5].

The incorporation of telemedicine into conventional healthcare encounters obstacles, notwithstanding its apparent benefits. These include matters of a technological and infrastructure nature as well as an ethical, legal, and security nature. Furthermore, telemedicine’s efficacy depends on healthcare providers’ acceptance of it and their effective use of it [6]. This is determined by their level of awareness, knowledge, and abilities in telemedicine technology and practices [7].

This study aims to evaluate telemedicine as it stands right now in a tertiary care environment from the standpoint of healthcare professionals. It investigates their awareness and understanding of telemedicine, their attitudes toward using it, their proficiency with telemedicine technologies, and the difficulties they encounter in implementing it. This evaluation is essential for finding knowledge and skill gaps, comprehending telemedicine usage obstacles, and figuring out medical professionals’ training requirements. These kinds of data are essential for formulating plans to improve telemedicine’s uptake and efficacy in healthcare systems.

## Materials and methods

Study design

This cross-sectional study was done to assess the awareness, knowledge, attitude, skills, and obstacles connected with telemedicine among doctors at Kurnool Medical College in Kurnool, Andhra Pradesh, India.

Study period and setting

The research was carried out over the course of 30 days in December 2022. The study was conducted at Kurnool Medical College, a well-known tertiary care facility in Kurnool, Andhra Pradesh, India.

Participants

The study comprised 100 physicians who work at Kurnool Medical College. They comprised doctors from internal medicine, pediatrics, surgery, dermatology, psychiatry, and emergency medicine, among other areas.

Data collection

A structured questionnaire was created and verified for the purposes of this investigation [8]. The questionnaire was divided into categories that evaluated the following:

Awareness and Knowledge of Telemedicine

Questions were designed to assess participants’ awareness of telemedicine technologies as well as their knowledge of telemedicine uses, benefits, and legal and ethical considerations.

Attitude Toward Telemedicine

The items evaluated the physicians’ attitudes, convictions, and willingness to incorporate telemedicine into their clinical practice.

Skills for Using Telemedicine Technologies

This section analyzed the participants’ experience with telemedicine platforms, self-reported skill levels, and technical ability.

Utilization Patterns

The questions were designed to obtain information about the frequency and types of telemedicine services used.

Challenges and Barriers

The questionnaire indicated perceived hurdles to telemedicine adoption, like infrastructure, data security, training, and patient acceptance.

The questionnaire was electronically distributed to all qualified doctors at the college. Every participant gave their informed consent before beginning their volunteer participation. The respondents’ confidentiality and anonymity were guaranteed (Appendix A).

Data analysis

The data collected from the structured questionnaire in this cross-sectional study were subjected to comprehensive analysis utilizing various statistical tools. Initially, descriptive statistics were employed to provide a succinct summary of the demographic characteristics of the participants, as well as their awareness, knowledge, attitudes, skills, and perceived barriers associated with telemedicine. For categorical variables such as specialty distribution and frequency of telemedicine usage, frequencies and percentages were computed to illustrate the distribution patterns. Moreover, inferential statistical analyses were conducted to delve deeper into the relationships between different variables. To assess the associations between demographic factors (such as age, gender, and specialty) and various aspects of telemedicine awareness, knowledge, attitudes, skills, and utilization patterns, appropriate inferential tests such as chi-square tests or Fisher’s exact tests were utilized. Furthermore, to explore potential predictors of favorable attitudes toward telemedicine or higher levels of telemedicine skills, multivariate analyses like logistic regression or linear regression models may have been employed, taking into account potential confounding variables. IBM SPSS Statistics for Windows, Version 23.0 (Released 2015; IBM Corp., Armonk, NY, USA) was used for the analysis.

Ethical considerations

The study received approval from Kurnool Medical College’s Institutional Ethics Committee (approval number IEC-KMC-GGH/181/2022). Every procedure carried out during the study complied with the Helsinki Declaration of 1964, its later amendments, and comparable ethical standards, as well as the institutional research committee’s ethical guidelines.

## Results

The study included 100 physicians from a tertiary care institution. Participants in the demographic breakdown, whose ages ranged from 28 to 60, were 60% male and 40% female. The majority of participants (70%) belonged to the 30- to 45-year age range. The representation came from a variety of specialties: emergency medicine, psychiatry, dermatology, and surgery made up 20% of the group, internal medicine made up 25%, and pediatrics made up 25% (Table 1).

**Table 1:** Participant demographics

Demographic feature	Percentage (%)
Gender (male)	60
Gender (female)	40
Age range (28-60 years)	100
Age group (30-45 years)	70
Specialty (internal medicine)	30
Specialty (pediatrics)	25
Specialty (surgery)	20
Other specialties	25

Awareness and knowledge of telemedicine

Awareness

Ninety-five percent of the participants said they were aware of telemedicine services and technology.

Knowledge

Seventy-five percent of the physicians accurately recognized its prospective uses and advantages in the provision of healthcare. Only 50% were aware of the legal and ethical concerns of telemedicine. Approximately 40% were aware of worldwide telemedicine rules and best practices (Table 2).

**Table 2:** Awareness and knowledge of telemedicine

Parameter	Percentage (%)
Awareness of telemedicine	95
Knowledge of applications and benefits	75
Familiarity with legal and ethical aspects	50
Awareness of international guidelines	40

Attitude toward telemedicine

Positive Attitude

Eighty percent of respondents had a favorable opinion of telemedicine’s use in clinical settings.

Perceived Benefits

Eighty-five percent agreed that telemedicine could considerably improve patient care, particularly in distant places.

Concerns

Twenty percent were concerned about the effectiveness and dependability of telemedicine services when compared to regular in-person consultations.

Future Outlook

Seventy-five percent of respondents were hopeful about telemedicine’s inclusion in future healthcare delivery systems (Table 3).

**Table 3:** Attitude toward telemedicine

Attitude aspect	Percentage (%)
Positive attitude	80
Belief in enhancing patient care	85
Concerns about effectiveness	20
Optimism about future integration	75

Skills for using telemedicine technologies

Experience Level

Sixty percent had prior experience using telemedicine platforms, and 70% of them rated their competence level as intermediate or advanced.

Lack of Experience

Forty percent reported a lack of training as their top reason for not using telemedicine.

Technical Proficiency

Only 30% were confident in troubleshooting technical issues using telemedicine software or equipment (Table 4).

**Table 4:** Skills for using telemedicine technologies

Skill aspect	Percentage (%)
Prior experience with platforms	60
Intermediate or higher skill level	70
Lack of training for nonusers	40
Confidence in technical troubleshooting	30

Utilization patterns

Frequency of Use

Among those who used telemedicine, 50% used it at least once each week.

Types of Services

Remote monitoring (30%) and video consultations (60%) were the services that were utilized most frequently.

Patient Demographics

The majority of physicians (65%) stated that they mostly used telemedicine for adult patients; they used it less frequently for patients who were younger or older (Table 5).

**Table 5:** Utilization patterns

Utilization aspect	Percentage (%)
Frequency of use (at least weekly)	50
Common services (video consultations)	60
Common services (remote monitoring)	30
Telemedicine use for adult patients	65

Training and educational needs

Training Demand

Ninety percent said there should be additional programs for education and training.

Interest in Workshops

A total of 85% expressed interest in telemedicine-related training classes and sessions.

Preferred Training Formats

The most popular training options were hands-on workshops (35%), and online courses (40%) (Table 6).

**Table 6:** Training and educational needs

Training aspect	Percentage (%)
Demand for more training	90
Interest in workshops	85
Preference for online modules	40
Preference for hands-on workshops	35

Challenges and barriers

Infrastructure Deficits

Sixty-five percent identified a lack of infrastructure as a significant hindrance.

Data Security Concerns

Fifty-five percent expressed concern regarding data security in telemedicine activities.

Training Shortages

Fifty percent identified inadequate training as a major problem.

Technical Problems

Forty-five percent of respondents mentioned connectivity issues as a challenge.

Patient Acceptance

Thirty percent of respondents reported that patient skepticism and acceptance were significant barriers to telemedicine use (Table 7).

**Table 7:** Challenges and barriers

Challenge/barrier	Percentage (%)
Lack of infrastructure	65
Data security concerns	55
Insufficient training	50
Technical problems (connectivity issues)	45
Patient acceptance issues	30

Future perspectives

Policy Development

Seventy percent of respondents emphasized the need for clearer policies and procedures to facilitate telemedicine integration.

Interdisciplinary Collaboration

Sixty percent of respondents indicated that interdisciplinary collaboration, which includes IT specialists, is essential for the development of telemedicine services (Table 8).

**Table 8:** Future perspectives

Perspective aspect	Percentage (%)
Need for clearer policies	70
Importance of interdisciplinary collaboration	60

## Discussion

The study conducted at Kurnool Medical College found that doctors have a high level of awareness (95%) and a good attitude (80%) toward telemedicine, indicating their preparedness to adopt digital healthcare modalities. This is consistent with the rising trend of healthcare digitization. However, there are significant gaps in knowledge, especially in the areas of telemedicine law and ethics (50%) and limited knowledge of international telemedicine guidelines (40%). This indicates the need for specialized educational programs targeted at improving physicians’ comprehension of the complex field of telemedicine [9].

The preference for video consultations (60%) and remote monitoring (30%) as telemedicine services, as well as the regular usage of these services by a significant proportion of doctors, demonstrates the rising integration of telemedicine into traditional healthcare delivery. This pattern is consistent with the worldwide shift toward increasingly digital health solutions [9].

Comparing the outcomes of this survey to those from other locations demonstrates significant variation in telemedicine awareness and attitudes. These disparities highlight the impact of diverse healthcare infrastructures, the accessibility of digital technologies, and the existence or nonexistence of training programs linked to telemedicine in diverse contexts. These variations highlight the need for region-specific telemedicine strategies that take into account the particular requirements and environments of each area [9-18].

For instance, studies from China [11], Saudi Arabia [10], and India [12] show varying levels of telemedicine readiness and receptivity, owing to disparities in healthcare systems and digital infrastructure. Similar studies have been conducted with healthcare professionals and students in a variety of countries, such as Saudi Arabia [13], Indonesia [14], Pakistan [15], and the United Arab Emirates [16]. These studies show the wide range of knowledge, perspectives, and readiness to use telemedicine services. This global viewpoint highlights how crucial it is to contextualize telemedicine adoption methods in order to maximize telemedicine’s potential to improve healthcare delivery and effectively address regional disparities [9-18].

Implications for practice

The study emphasizes the importance of comprehensive training programs, as expressed by 90% of respondents. To address the 40% of respondents who stated that a lack of training was a barrier, training can improve abilities and confidence in the use of telemedicine. Furthermore, for telemedicine to be used more widely, infrastructure deficiencies (65%) and data security issues (55%) must be addressed.

The results of the study also point to the necessity of developing policies, with 70% of participants citing the need for more precise guidelines. This is in line with the worldwide demand for strong telemedicine regulations to guarantee safe, moral, and efficient telehealth procedures.

Limitations

The study’s limitations include a focus on a single tertiary care hospital, which may restrict the findings’ generalizability. Furthermore, the study’s cross-sectional design offers a snapshot viewpoint that might not accurately reflect changing attitudes toward telemedicine.

Future research

Additional research is needed to investigate long-term changes in telemedicine perceptions among healthcare providers. A more thorough understanding of the effects of telemedicine would come from research on patient outcomes and satisfaction in the environment of telemedicine, as well as from comparative studies across other healthcare settings.

## Conclusions

This study underscores the promising trajectory of telemedicine integration within healthcare delivery, indicating a favorable inclination among physicians toward its adoption. Observations regarding telemedicine utilization trends reveal a notable integration into clinical practice, particularly evident in the increasing reliance on video consultations and remote monitoring modalities. Moreover, the study accentuates the pressing need to surmount infrastructural inadequacies, mitigate data security concerns, and bridge training deficiencies to fully harness telemedicine’s potential. Consequently, the study underscores telemedicine’s transformative capacity within healthcare delivery, advocating for concerted efforts to address challenges and optimize its implementation for enhanced patient care and clinical outcomes.

## Appendices

Appendix A

Telemedicine Awareness and Knowledge

1. Are you aware of telemedicine services and technologies?

Yes

No

2. Which of the following uses and benefits of telemedicine are familiar to you? (Select all that apply.)

Remote patient monitoring

Video consultations

Electronic prescriptions

Access to healthcare in rural areas

3. Are you aware of the legal and ethical considerations surrounding telemedicine?

Yes

No

4. Do you know about international telemedicine regulations and best practices?

Yes

No

Attitude Toward Telemedicine

1. How would you describe your overall perception of telemedicine?

Very favorable

Somewhat favorable

Neutral

Somewhat unfavorable

Very unfavorable

2. Do you believe telemedicine, particularly in rural locations, can greatly enhance patient care?

Strongly agree

Agree

Neutral

Disagree

Strongly disagree

3. Are you concerned about the efficacy and reliability of telemedicine services versus in-person consultations?

Yes

No

4. Are you optimistic about the integration of telemedicine into future healthcare delivery models?

Very optimistic

Optimistic

Neutral

Pessimistic

Very pessimistic

Skills for Using Telemedicine Technologies

1. Do you have experience with telemedicine platforms?

Yes

No

2. How would you rate your skill in using telemedicine technology?

Beginner

Intermediate

Advanced

Expert

3. If you have not used telemedicine, what is the main reason?

Lack of training

Lack of access

Not interested

Other (please specify)

Utilization Patterns

4. How frequently do you utilize telemedicine services?

Daily

Weekly

Monthly

Rarely

Never

5. Which telemedicine service categories do you use most regularly? (Select all that apply.)

Remote monitoring

Video consultations

Electronic prescriptions

Other (please specify)

Training and Educational Needs

6. Do you think there should be more telemedicine-related training programs and educational opportunities?

Strongly agree

Agree

Neutral

Disagree

Strongly disagree

7. What kind of telemedicine training are you most interested in? (Select all that apply.)

Hands-on workshops

Online courses

Webinars

In-person seminars

Challenges and Barriers

8. What are the primary barriers preventing your practice from implementing telemedicine? (Select all that apply.)

Infrastructure deficits

Data security concerns

Lack of training

Connectivity issues

Patient skepticism and acceptance

Other (please specify)
